# Intimate partner violence during pregnancy among married women in Putalibajar municipality, Nepal

**DOI:** 10.3389/fpsyg.2023.1158406

**Published:** 2023-06-02

**Authors:** Rakshya Sharma, Hari Prasad Kaphle

**Affiliations:** School of Health and Allied Sciences, Pokhara University, Pokhara, Nepal

**Keywords:** intimate partner violence, pregnancy, postpartum woman, factors associated, cross sectional study

## Abstract

**Introduction:**

Intimate partner violence during pregnancy (IPVDP) is increasingly being recognized as a significant problem in the developing world due to its adverse health consequences on both pregnant women and children. The objective of the study is to measure the magnitude of intimate partner violence during pregnancy and the factors associated with IPVDP.

**Methods:**

A community-based cross-sectional study was conducted among 263 married women in their extended postpartum period between October 2019 and March 2020 in Putalibajar municipality, Nepal. A face-to-face interview was conducted and data were collected using an interview schedule. A Chi-square test and logistic regression analysis were performed to examine the association between IPVDP and the independent variables.

**Results:**

Among the 263 women interviewed, 30% experienced IPV during pregnancy, the most common type of violence was controlling behavior (20.2%) followed by emotional (18.6%), sexual (10.6%), economic (6.1%), and physical violence (5.3%). It was observed that IPV was more likely to occur among women whose husbands consumed alcohol (AOR = 3.171; CI 95%: 1.588–9.167), women whose husbands consumed tobacco (AOR =3.815; CI 95%: 2.157–7.265), women who sometimes received family support during pregnancy (AOR =2.948; CI 95%: 1.115–7.793) and women who did not decide on marriage timing (AOR =2.777; CI 95%: 1.331–5.792).

**Conclusion:**

Three out of ten pregnant women experienced IPVDP. To prevent violence, and ensure women’s empowerment, formulating strict laws and discouraging the element of a violent milieu is important.

## Introduction

Intimate partner violence (IPV) is defined as any act of physical, sexual, or psychological controlling actions or economic violence that takes place between intimate partners. It is regarded as global public health and human rights issue (García-Moreno et al., 2005). Globally, 30% of women experience violence by their intimate partner (García-Moreno et al., 2013). In recent years, intimate partner violence during pregnancy (IPVDP) is increasingly being recognized as a significant problem in the world due to its prevalence and its adverse health consequences on both pregnant women and children. The World Health Organization (WHO) estimated that, globally, the proportion of ever-pregnant women who reported violence during pregnancy varied from 1 to 28%. Physical violence exceeded 5% in 11 out of 15 settings where one-half of the women reported being punched or kicked in the abdomen and the majority (90%) of abuse was perpetrated by the biological father of the child she was carrying (García-Moreno et al., 2005).

Women experiencing violence during pregnancy are less likely to receive prenatal care and may experience adverse health outcomes such as poor nutrition, miscarriage, premature labor, trauma, placenta abruption, low birth weight (LBW), stillbirth, UTI, Chronic Pelvic infection, depression, and maternal death (García-Moreno et al., 2005; Nejatizade et al., 2017; Regmi et al., 2017). Nepal is a diverse ecological, ethnic, cultural, and multi-language society but is predominantly a Hindu country where it has its own cultural practices, norms, and values regarding men and women. In the context of Nepal, the key structural factors that make women vulnerable to IPV are economic dependency on men, cultural obligations, early marriage, giving birth to a girl, dowry practices, and lower social position which constructs and reinforces male dominance in society (Office of the Prime Minister and Council of Ministers, 2012; Deuba et al., 2016; Gurung and Acharya, 2016).

According to the Nepal Demographic Health Survey (NDHS) 2016, 26% of ever-married women have ever experienced physical, sexual, or emotional violence at the hands of their husbands. Of the 6% of women who have ever been pregnant and experienced physical violence during pregnancy, the majority of them experienced violence that was perpetrated by their current husbands (Ministry of Health Nepal, 2016). Studies conducted in Nepal showed a significant prevalence of IPVDP, 28.9% in Dhanusha and 53.2% in Kathmandu (Regmi et al., 2017; Singh et al., 2018). The aim of the study was to identify the magnitude of IPVDP and the factors associated with IPVDP.

## Methodology

### Study design and population

A community-based cross-sectional analytical study was carried out among 263 married women aged 15 to 49 years who had at least one child aged 12 months and younger in Putalibajar municipality, Gandaki Province, Nepal from October 2019 to March 2020. The sample size was calculated by using the finite population formula:

$n=\frac{Z_{\propto }^{2}pqN}{d^{2}\left(N-1\right)+Z_{\propto }^{2}pq}\text{.}$

A sample size of 263 was determined based on a 28.9% prevalence of IPVDP from a previous study conducted in Dhanusha, Nepal, with a 10% non-response rate and an estimated population of children aged 0–11 months in 2075/76 (2018/2019) of Putalibajar municipality (N) of 954 (Singh et al., 2018). Married women aged 15 to 49 years who had at least one child aged 12 months or younger were eligible for the study but mothers who were temporary residents, who refused to participate, who had no contact with their husband during the period of pregnancy, who could not answer, and who did not understand the Nepali language were excluded. A multi-stage random sampling technique was followed where 4 out of 14 wards were selected using a simple random technique; participants from each selected ward were determined proportionately based on the sample population. Finally, a systematic random sampling technique was carried out to select individual study participants where the sampling interval (K) was determined by dividing the total population size by the sample size.

### Data collection and quality control

A face-to-face interview was carried out using an interview schedule to gather the information. To ensure the validity of the study, a tool was developed by using a standard questionnaire on IPVDP from the instrument of the WHO multi-country study questionnaire (García-Moreno et al., 2005). The Cronbach’s alpha values for the physical and psychological IPV scales of the WHO multi-country study questionnaire were 0.78 and 0.75, respectively, in a study conducted in Bangladesh (Islam et al., 2017). Reliability was ensured by pretesting the tool among 10% of the estimated sample size. The tool was developed in both English and Nepali language.

### Variables

The interview schedule included questions about socio-demographic characteristics such as family type, ethnicity, religion, the main occupation of the family, the woman’s age, education, and involvement in an income-generating activity. The participants also provided information about their partner’s characteristics such as age, education, involvement in an income-generating activity, substance use, gambling, presence of polygamy, and partner’s preference for the specific sex of their child. Obstetric and reproductive characteristics such as age at marriage, type of marriage, the decision on marriage timing, duration of the marriage, gravida, previous history of miscarriage or induced abortion, number of living children, pregnancy intention, family support, and timing of 1^st^ antenatal care (ANC) visit.

The dependent variable (IPVDP) was measured through a structured questionnaire regarding physical, sexual, emotional, and economic violence and controlling behavior from the instrument of the WHO multi-country study (García-Moreno et al., 2005). Participants answered yes or no whether they have experienced each of five forms of IPV.

### Data analysis

Data entry was done using EPI-DATA and data analysis was done using SPSS. Descriptive statistics were used to describe the study population, which included means, ranges, frequencies, and percentages. The first step in the analysis was to find out the association between IPVDP and independent variables using the chi-square test. Variables that were significant in the chi-square test with a value of *p* < 0.05 were further analyzed by using bivariate and multivariate logistic regression to assess the strength of the association.

### Ethical consideration

The study was given ethical approval by the Nepal Health Research Council (NHRC). The 2001 guidelines of Ethical and Safety Recommendations for Research on Domestic Violence against Women by the WHO were followed (World Health Organization, 2001). The participants were fully informed about the nature and benefits of the research and full informed consent was taken prior to the conduction of the interview. The participants were informed about the rights of women under the law regardless of their experience of violence. If a woman needed help, contact was made with support institutions or police stations only after the woman’s consent had been obtained.

## Results

### Socio-demographic characteristics

Out of 263 participants, more than half of the respondents [146 (55.5%)] were between 25 and 34 years old. Of the upper caste group 117(44.5%) belonged to Hindu 228(86.7%). The mean age of the participants was 27 years (SD ± 4.9). Nearly all respondents were literate [256 (97.3%)], and nearly half [125(47.5%)] of the participants belonged to a nuclear family. The majority of the participants’ husbands were aged between 25 and 34 years [168(63.9%)]. The mean age of the participants’ husbands was 30 years old (SD ± 5.4), and ranged from 19 to 51 years. Almost all the participants’ husbands were literate [261(99.2%)] and were involved in any type of income-generating activity [246(93.5%); Table 1].

**Table 1:** Socio-demographic characteristics.

Variable	Frequency (*n*)	Percent (%)
Woman’s age		
15–24	92	35
25–34	146	55.5
≥ 35	25	9.5
Family type		
Nuclear	125	47.5
Joint	115	43.7
Extended	23	8.7
Religion		
Hinduism	228	86.7
Buddhism	8	3.0
Muslim	25	9.5
Christianity	2	0.8
Ethnicity		
Upper caste group	117	44.5
Relatively advantaged Janajati	48	18.3
Disadvantaged Janajati	23	8.7
Religious minorities	25	9.5
Dalit	50	19.0
Woman’s education		
No formal education	7	2.7
Primary	17	6.5
Secondary	105	39.9
Higher Secondary	93	35.4
Bachelor and above	41	15.6
Involved in income-generating activity		
Involved	73	27.8
Not involved	190	72.2
Husband’s age		
≤24	25	9.5
25–34	168	63.9
≥35	70	26.6
Husband’s education		
No formal education	2	0.8
Primary	6	2.3
Secondary	132	50.2
Higher Secondary	70	26.6
Bachelor and above	53	20.2
Husband’s involvement in income-generating activity		
Involved	246	93.5
Not involved	17	6.5

### Magnitude of IPVDP

Out of 263 participants, 79 (30%) experienced any kind of IPVDP. The most common type of IPV faced by the participants among the different forms of violence was controlling behavior [53(67.1%)], followed by emotional [49(62.0%)], sexual [28(35.4%)], economic [16(20.3%)], and physical violence [14(17.7%)].

### Association between variables and IPVDP

#### Association between socio-demographic characteristics and IPVDP

Intimate partner violence during pregnancy was significantly associated with family size (*p* < 0.01), ethnicity (p < 0.01), the main occupation of the family (*p* < 0.05), and women’s education (*p* < 0.05; Table 2).

**Table 2:** Association between socio-demographic characteristics of participants and Intimate partner violence during pregnancy (IPVDP).

Variable	IPVDP		Total	Value of *p*
	Yes 79(30.0%)	No 184 (70.0%)		
Family type				
Nuclear	26(20.8%)	99(79.2%)	125	0.008**
Joint	44(38.3%)	71(61.7%)	115	
Extended	9(39.1%)	14(60.9%)	23	
Religion				
Hinduism	67(29.4%)	131(70.6%)	228	0.556
Others	12(34.3%)	23(65.7%)	184	
Ethnicity				
Upper caste group	30(25.6%)	87(74.4%)	117	0.002**
Relatively advantaged Janajati	7(14.6%)	41(85.4%)	48	
Disadvantaged Janajati	8(34.8%)	15(65.2%)	23	
Religious Minorities	9(36.0%)	16(64.0%)	25	
Dalit	25(30.0%)	25(70.0%)	50	
Main occupation of the family				
Agriculture	5(41.7%)	7(53.3%)	12	0.030*
Job	13(16.7%)	65(83.3%)	78	
Business	28(42.4%)	38(57.6%)	66	
Daily wage laborer	7(31.8%)	15(68.2%)	22	
Foreign laborer	9(33.3%)	18(66.7%)	27	
Foreign Service	17(29.3%)	41(70.7%)	58	
Woman’s age				
< 25	27(29.3%)	65(70.7%)	92	0.967
25–34	44(30.1%)	102(69.9%)	146	
≥ 35	8(32.0%)	17(68.0%)	25	
Woman’s education				
< 10 years of schooling	26(40.6%)	38(59.4%)	64	0.027*
≥ 10 years of schooling	50(26.0%)	142(74.0%)	192	
Woman’s involvement in any income-generating activity				
Involved	23(31.9%)	49(68.1%)	72	0.679
Not involved	56(29.3%)	135(70.7%)	191	

Other religion = Buddhism, Islam, and Christianity. *p-value significant at *α* <0.05, **p-value significant at *α* <0.01.

#### Association between the participants’ husband’s characteristics and IPVDP

IPVDP was strongly associated with the husband’s alcohol consumption, frequency of alcohol consumption, and the husband’s tobacco consumption (*p* < 0.001). Similarly, family support during pregnancy and the husband’s involvement in gambling were also associated with IPVDP (*p* < 0.01; Table 3).

**Table 3:** Association between participants’ husband’s characteristics and IPVDP.

Variable	IPVDP		Total	Value of *p*
	Yes 79(30.0%)	No 184(70.0%)		
Husband’s age				
≤ 24	7(28%)	18(72%)	25	0.775
25–30	53(31.5%)	115(68.5%)	168	
≥ 35	19(27.1%)	51(72.9%)	70	
Husband’s education				
< 10 years of schooling	21(36.8%)	36(63.2%)	57	0.222
≥ 10 years of schooling	58(28.4%)	146(71.6%)	204	
Husband’s involvement in income-generating activities				
Involved	73(29.7%)	173(70.3%)	246	0.625
Not involved	6(35.3%)	11(64.7%)	17	
Husband’s alcohol consumption				
Yes	52(44.4%)	65(55.6%)	117	<0.001***
No	27(18.5%)	119(81.5%)	146	
Frequency of alcohol consumption				
Daily	18 (90%)	2 (10%)	20	<0.001***
Usually	11(47.8%)	12 (52.2%)	23	
Sometimes	17 (31.5%)	37 (68.5%)	54	
Rarely	6 (30%)	14 (70%)	20	
Husband’s tobacco consumption				
Yes	32(54.2%)	27(45.8%)	59	<0.001***
No	47(23%)	157(77%)	204	
Presence of gambling				
Yes	28(45.9%)	33(54.1%)	61	0.002**
No	51(25.2%)	151(74.8%)	202	
Polygamy^#^				
Yes	4(33.3%)	8(66.7%)	12	1.000
No	75(29.9%)	176(70.1%)	251	
Husband’s preference for a specific sex of the child				
No preference	54(27%)	146(73%)	200	0.122
Preference for son	16(43.2%)	21(56.8%)	37	
Preference for daughter	9(34.6%)	17(65.4%)	26	
Family support				
Daily	13(17.3%)	62(82.7%)	75	0.008**
Usually	21(26.9%)	57(73.1%)	78	
Sometimes	32(38.1%)	52(61.9%)	84	
Rarely	8(47.1%)	9(52.9%)	17	
Never	5(55.6%)	4(44.4%)	9	

# Yates continuity correction. **p-value significant at *α* <0.01, ***p-value highly significant at *α* <0.001.

#### Association between obstetric and reproductive characteristics and IPVDP

IPVDP was significantly associated with decisions about marriage timing by the participants (*p* < 0.01) and family support during pregnancy (*p* < 0.001; Table 4).

**Table 4:** Association between IPVDP and obstetric and reproductive characteristics.

Variable	IPVDP		Total	Value of *p*
	Yes 79(30.0%)	No 184(70.0%)		
Woman’s age at marriage				
≤ 19	42(33.3%)	84(66.7%)	126	0.264
≥ 20	37(27%)	100(73%)	137	
Type of marriage				
Love marriage	28(29.5%)	67(70.5%)	95	0.881
Arranged marriage	51(30.4%)	117(69.6%)	168	
Duration of marriage				
≤ 4	31(30.4%)	71(69.6%)	102	0.634
5–9	25(26.9%)	68(73.1%)	93	
≥ 10	23(33.8%)	45(66.2%)	68	
Gravida				
Primigravida	33(28.0%)	85(72.0%)	118	0.508
Multigravida	46(31.7%)	99(68.3%)	145	
Parity				
Nulliparous	37(29.1)	90(70.9)	127	0.953
Primiparous	30(30.9)	67(69.1)	97	
Multiparous	12(30.8)	27(69.2)	39	
Total number of living children excluding their recent child (n = 137)				
1	70(70.0%)	30(30.0%)	100	0.565
> 1	24(64.9%)	13(35.1%)	37	
Presence of abortion/miscarriage				
Yes	17(40.5%)	25(59.5%)	42	0.107
No	62(28.1%)	159(71.9%)	221	
Pregnancy intention				
Intended	45(26.5%)	125(73.5%)	170	0.088
Unintended	34(36.6%)	59(63.4%)	93	
Timing of first antenatal visit				
Early initiation of ANC	74(29.7%)	175(70.3%)	249	0.860
Late initiation of ANC	5(35.7%)	9(64.3%)	14	
Decision on marriage timing				
Yes	38(22.4%)	132(77.6%)	170	< 0.001***
No	41(44.1%)	52(55.9%)	93	

***p-value highly significant at *α* <0.001.

#### Bivariate and multivariate logistic regression analysis between IPVDP and selected variables

In multivariate logistic regression analysis, after adjusting for potential confounding factors, IPV was significantly associated with the husband’s alcohol consumption (AOR = 3.171; CI 95%:1.454 – 6.912), husband’s tobacco consumption (AOR = 3.815; CI 95%:1.588 – 9.167). Similarly, women who reported receiving family support only sometimes during their pregnancy were more likely to experience IPV in comparison to women who received family support on a daily basis (AOR = 2.948; CI 95%:1.115–7.793), and women who did not decide upon their marriage timing (AOR = 2.777; CI 95%:1.331–5.792) were more likely to experience IPVDP (Table 5).

**Table 5:** Bivariate and multivariate logistic regression analysis between IPVDP and selected variables.

Variable	UOR(95%CI)	AOR(95%CI)
Family type		
Nuclear	1	1
Joint	2.360(1.331–4.184)	1.934(0.864–4.326)
Extended	2.448(0.954–6.280)	1.465(0.421–5.097)
Ethnicity		
Upper caste group	1	1
Relatively advantaged Janajati	0.495(0.201–1.221)	0.258(0.086–0.778)
Disadvantaged Janajati	1.547(0.596–4.012)	1.041(0.257–4.207)
Religious Minorities	1.631(0.653–4.077)	1.088(0.326–3.628)
Dalit	2.9(1.451–5.797)	1.871(0.711–4.923)
Main occupation of the family		
Agriculture	1.723(0.479–6.192)	1.555(0.346–6.983)
Job	0.482(0.212–1.096)	0.349(0.116–1.053)
Business	1.777(0.842–3.751)	1.713(0.674–4.356)
Daily wage laborer	1.125(0.390–3.250)	0.087(0.015–0.515)
Foreign laborer	1.206(0.453–3.212)	0.641(0.184–2.239)
Foreign Service	1	1
Woman’s education		
<10 years of schooling	1.943(1.073–3.519)	2.168(0.884–5.316)
≥10 years of schooling	1	1
Husband’s alcohol consumption		
Yes	3.526(2.025–6.139)	**3.171(1.454–6.912)**
No	1	1
Husband’s tobacco consumption		
Yes	3.959(2.157–7.265)	**3.815(1.588–9.167)**
No	1	1
Presence of gambling		
Yes	2.512(1.385–4.556)	1.736(0.758–3.973)
No	1	1
Family support		
Daily	1	1
Usually	1.757(0.806–3.832)	1.808(0.646–5.056)
Sometimes	2.935(1.397–6.167)	**2.948(1.115–7.793)**
Rarely	4.239(1.377–13.051)	1.916(0.474–7.745)
Never	5.962(1.406–25.271)	4.497(0.680–29.757)
Decision on marriage timing		
Yes	1	1
No	2.739(1.587–4.727)	**2.777(1.331–5.792)**

^*^Value of *p* significant at *α* < 0.05, ^**^Value of *p* significant at *α* < 0.01, ^***^Value of *p* highly significant at *α* < 0.001.

Adjusted for family type, ethnicity, main occupation of the family, women’s education, husband’s alcohol consumption, husband’s tobacco consumption, gambling, family support, and decision on marriage timing. Bold values indicate significant result after adjustment of covariates.

## Discussion

The prevalence of IPVDP in this study was 30% among the 263 participants, which is consistent with another study from Dhanusha, Nepal, showing a prevalence of 28.9%. This may be due to the similarities in the population based on the setting (Singh et al., 2018). The prevalence figures of this study were also consistent with the global prevalence of IPVDP estimated by the WHO in a multi-country study (García-Moreno et al., 2005).

However, our prevalence figure is lower than that of other studies conducted in Ethiopia (Abebe Abate et al., 2016), Nigeria (Ezeudu et al., 2019), Vietnam (Hoang et al., 2016), Kenya (Makayoto et al., 2013), Rwanda (Ntaganira et al., 2008), South Korea (Lee and Lee, 2018), and Gambia (Idoko et al., 2015) but higher than the studies conducted in Belgium (Van Parys et al., 2014) Japan (Doi et al., 2019), South Africa (Groves et al., 2012), Guatemala (Johri et al., 2011), and India (Jain et al., 2017). Such a difference may be attributed due to the differences in methodologies, population, and assessments that have been used in the previous research. A strong association was found between alcohol use by the husband and the experience of IPVDP by women. Consistent with our findings, strong links have been found between alcohol use and the occurrence of IPV in many countries such as India (Das et al., 2013), Kenya (Makayoto et al., 2013; Owaka et al., 2017), Rwanda (Ntaganira et al., 2008), Brazil (Teixeira et al., 2015), and Zimbabwe (Shamu et al., 2013).

Our study showed that women whose husbands consumed alcohol were more than three times more likely to experience violence than women whose husbands did not consume alcohol. Studies from other countries showed that women who had partners who drank alcohol were more than two times as likely in Kenya (Makayoto et al., 2013), and four times as likely in Rwanda (Ntaganira et al., 2008), to experience violence. This might be explained due to the low-socio-economic status of the family, societal beliefs, or the impulsive personality of the husband, which accounts for an unhappy and stressful partnership, thus resulting in violence. However, our study does not determine that the increased stress from the use of alcohol leads to IPV or that the abuse of alcohol is a result of the stress of IPV but the association is significant.

Women whose husbands consumed tobacco were more than three times more likely to experience violence than women whose husbands did not consume tobacco. Similar to our finding, a study conducted in Iran showed an association between a smoking partner and psychological violence during pregnancy where women whose partner smoked were more than two times more likely to experience violence during pregnancy (Hajikhani Golchin et al., 2014). However, this study took only one type of violence to show their association while in our study, the association was shown with the experience of any type of violence as a whole.

A significant association was found between family support and IPVDP. Our study revealed that women who never received any assistance from family members in taking care of them during pregnancy or helping with household chores were almost six times more likely to experience violence during pregnancy. Similar literature on this topic was not found in previous studies. However, this variable is very important since pregnancy is a time when women need more care and support from their families. No support during this period might cause quarreling and violence in the family. However, our study does not determine that a lack of care during pregnancy led to the generation of a stressful relationship between the woman and their intimate partner or that the experience of IPV led to a lack of care during pregnancy but there remains a strong association.

Similarly, a strong association was found between the woman’s decision on marriage timing and the experience of IPVDP. Our study showed that women who did not decide upon their marriage timing were more than two times more likely to experience IPVDP.

## Conclusion

Our results demonstrate that in Putalibajar municipality, Syangja, three out of ten women experienced IPVDP. Controlling behavior inflicted by the husband was the most common type of violence followed by emotional, sexual, economic, and physical violence. To prevent violence, educating young people about respectful relationships, ensuring women’s empowerment by focusing on higher education opportunities as well as discouraging the element of a violent milieu are important. Appropriate laws prohibiting violence against women and actions regarding screening of IPVDP at antenatal visits with proper management and referral to relevant care are needed.

## Data availability statement

The raw data supporting the conclusions of this article will be made available by the authors, without undue reservation.

## Ethics statement

The studies involving human participants were reviewed and approved by IRC, Pokhara University Research Center and ERB, Nepal Health Research Council. The patients/participants provided their written informed consent to participate in this study.

## Author contributions

RS and HPK: conceptualization, data curation, formal analysis, methodology, visualization, and writing—review and editing. RS: funding acquisition, investigation, and writing—original draft. HPK: supervision and validation. All authors contributed to the article and approved the submitted version.

## Funding

This research has been funded by Nepal Health Research Council with Under Graduate Health Grant.

## Conflict of interest

The authors declare that the research was conducted in the absence of any commercial or financial relationships that could be construed as a potential conflict of interest.

## Publisher’s note

All claims expressed in this article are solely those of the authors and do not necessarily represent those of their affiliated organizations, or those of the publisher, the editors and the reviewers. Any product that may be evaluated in this article, or claim that may be made by its manufacturer, is not guaranteed or endorsed by the publisher.
